# The Failure Envelope Concept Applied To The Bone-Dental Implant System

**DOI:** 10.1038/s41598-017-02282-2

**Published:** 2017-05-17

**Authors:** R. Korabi, K. Shemtov-Yona, A. Dorogoy, D. Rittel

**Affiliations:** Faculty of Mechanical Engineering, Technion, 32000 Haifa, Israel

## Abstract

Dental implants interact with the jawbone through their common interface. While the implant is an inert structure, the jawbone is a living one that reacts to mechanical stimuli. Setting aside mechanical failure considerations of the implant, the bone is the main component to be addressed. With most failure criteria being expressed in terms of stress or strain values, their fulfillment can mean structural flow or fracture. However, in addition to those effects, the bony structure is likely to react biologically to the applied loads by dissolution or remodeling, so that additional (strain-based) criteria must be taken into account. While the literature abounds in studies of *particular* loading configurations, e.g. angle and value of the applied load to the implant, a *general* study of the admissible implant loads is still missing. This paper introduces the concept of failure envelopes for the dental implant-jawbone system, thereby defining admissible combinations of vertical and lateral loads for various failure criteria of the jawbone. Those envelopes are compared in terms of conservatism, thereby providing a systematic comparison of the various failure criteria and their determination of the admissible loads.

## Introduction

In the year 1952, Branemark defined the term osseointegration as the direct functional and structural connection between the living bone tissue and titanium implant surface^1^. Today, the high success rate of osseointegration depends largely on the integrity and nature of the *bone-implant interface*. This interface is actually created once the implant is inserted into the bone, and its main role is to bear and transfer functional mastication loads to the surrounding bone.

If one sets aside the mechanical response of the much stronger dental implant, the jawbone is the component of the system that is likely to deform (flow), or even fracture, according to the applied loads, just like any mechanical structure. For plastic flow and fracture, classical failure criteria have long been available in the field of Materials Mechanics. Those criteria are generally formulated in terms of stresses or strains^2^.

However, in addition to flow or fracture, the living bone tissue can be regarded as a self-optimizing structure that adapts to exogenous load conditions^3^. According to Frost’s “mechanostat” theory^4, 5^, the biological response of bones depends on the level of strain exerted. More precisely, a recent study by Piccinini *et al*.^6^ identified the octahedral shear (equivalent) strain as the most relevant strain for the mechanostat theory.

The mechanostat identifies five main ranges of strain, namely:

- Disuse (below <1000 µɛ) leading to bone atrophy.
- Adapted state (between 1000 µɛ–1500 µɛ), for which bone homoeostasis is maintained.
- Physiological overload (between 1500 µɛ–3000 µɛ), where bone modeling takes place, leading to increased bone mass due to the physiologic demand.
- Pathologic overload (above 3000 µɛ) that leads to bone damage and absorption.
- For strain levels in excess of 25000 µɛ, the ultimate bone strength is reached, resulting in catastrophic fracture.

It is well known that the load transfer from the implant to the host bone induces stress and strain fields around the implant, that are mediated by the interface. The magnitude of these fields is affected by a number of biomechanical factors, such as type of loading, type of rehabilitation, quantity and quality of the surrounding bone, implant material and mechanical properties of the system components^7^.

Loss of osseointegration can be related to overloading of the bone, primarily due to improper occlusion, prosthesis and/or implant design. The high stresses create a large strain field in the pathological area, that is sufficient to stimulate biological bone resorption, thereby jeopardizing the implant’s long term stability^7^.

Many *clinical* studies, both *in vivo* and *in vitro*, have attempted to evaluate the overload effect on the bone around implants in order to optimize implant treatment, and prevent loss of osseointegration due to excessive use^8^. Unfortunately, no clear conclusion was reached from these studies. While some studies reported bone loss due to heavy occlusal contacts^9–12^, others reported only a minor effect of the static and dynamic forces on the surrounding bone^13–15^. Isidor *et al*.^11^ performed an animal study to assess the effect of load direction on implant failure after 18 month of lateral force. About 65% of the implants lost osseointegration radiographically and histologically. However, ensuing studies failed to confirm this effect^16^.

It may be argued that in those clinical works, the load magnitude and the exact bone stresses were not accurately determined, which might explain this controversy in the biomechanical field.

The direct measurement of the stress-strain distribution around loaded implants is a highly delicate task, mainly because of the limited ways to measure and appraise those fields within a living structure. However, the existing computational methods, that simulate the mechanical behavior of bone and bone-implant interface, can bring a valuable insight into the processes that govern bone reactions, and therefore provide guidelines towards the optimization of clinical treatments to obtain long term success of dental implants. Finite element analysis (FE) is the most popular approach for mechanical simulations, and it has been widely used in the biomechanical field. Despite the powerful and effective calculation capabilities of this method, its overall accuracy depends on the accurate definition of the model geometry, material properties, boundary conditions and interactions between components.

The difficulty in modeling bone tissue response to loads lies both in its complex geometry and components mechanical properties. The mechanical properties of the cortical bone were found to be highly regional and anisotropic^17^, leading sometimes to modeling simplifications, some of which may be questionable. For example, it was found that a wrong identification of the principal axes of the cortical bone may lead to the wrong determination of its mechanical properties^17, 18^.

For the trabecular bone, it is even more difficult to accurately determine its mechanical properties, as inherent in porous materials. Lakatos *et al*.^19^ recently determined the Young’s modulus of trabecular bone obtained from fresh human cadaver’s mandible. Their compression tests were combined with numerical simulations. The trabecular bone Young’s modulus values were reported to range from 6.9 to 199.5 MPa, which is quite large.

In parallel, a wealth of studies was performed to simulate and calculate the functional loads’ effect on teeth and implants using FE modeling, both 2 and 3-dimensional. Most of them referred to the living bone as an isotropic material without explicit consideration of the nature of the bone-implant interface, so that perfect bonding conditions have often been assumed. In addition, no systematic study has been performed yet, to analyze the individual influence of the vertical and lateral load components on the bone fields. As of today, most studies assumed various combinations of arbitrary load magnitudes and loading angle, thereby precluding establishment of general conclusions (see e.g. refs 20–23). On a more general note, Natali *et al*.^24^ evaluated the application of FE models in implant dentistry, and characterized the bone-implant interaction. In their work, the authors varied the bone stiffness, as the bone’s elastic modulus increases over time as a result of post-operative healing process. In addition, this study evaluated the stress/strain fields on the peri-implant tissue as a result of load orientation and showed that the lateral displacement of the implant at the coronal level increases as the force angle increases. No reference to bone yielding was given. Even though, the study clearly showed the effect of the study parameters and loading conditions on the biomechanical response, and emphasized the importance of model accuracy and validation.

From this brief literature survey, it appears that accurate information is still missing regarding the influence of the bone model characteristics, interfacial conditions, and admissible load levels (both lateral and vertical), all being inter-related through the selection of a failure criterion. This information is precisely what is needed to assess the successful bio-integration of an implant into the host bone.

Therefore, this paper will focus on the determination of admissible load levels for the bone implant interface, based not only on strength criteria, such as bone yield stress, maximum compression strain and implant lateral displacement, but also on the admissible octahedral shear strain levels to include bone microstructural evolutions. The admissible load levels will be expressed as “failure envelopes”, so that the degree of conservatism of each criterion can be appraised.

## Results

Three central parameters, which altogether define the numerical model presented here, were considered, as follows:

- The mechanical properties of the cortical bone, namely a comparison between isotropic and anisotropic cortical bone models, together with an isotropic trabecular bone.
- The effect of the coefficient of friction (COF) between the implant and the bone.
- The separate and the combined effect of lateral loading (buccal-lingual, BL direction) versus vertical loading (apical-coronal, AC direction) on cortical bone yielding. It is worth mentioning here that for all stress based criteria examined in this work, the cortical bone always yielded before the trabecular bone.

**Figure 1:**
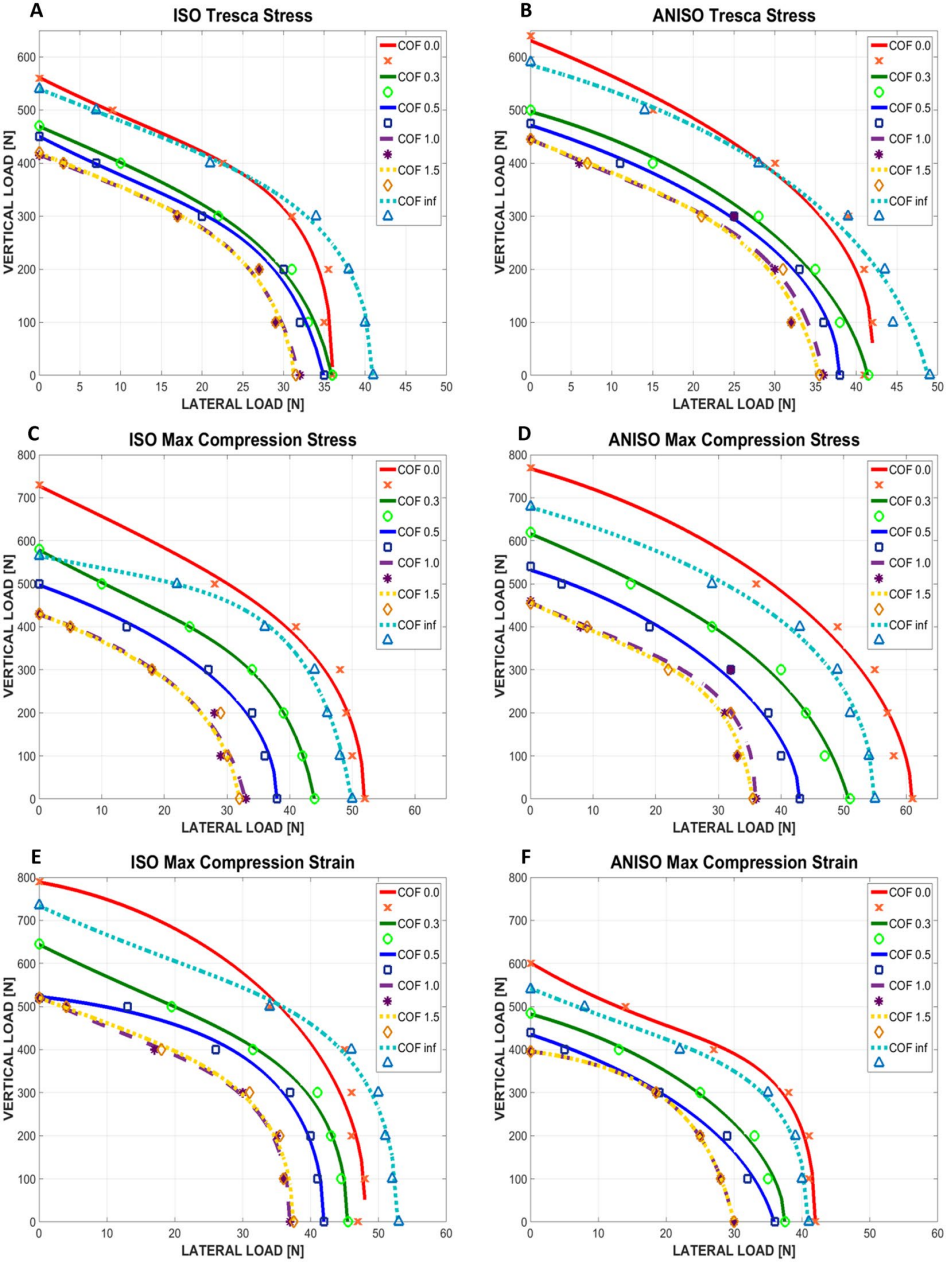
Graphs of lateral vs. vertical load causing cortical bone yielding. (**A**,**B**) Isotropic and anisotropic cortical bone respectively, for Tresca stress yield criterion. (**C**,**D**) Isotropic and anisotropic cortical bone respectively, for maximum compression stress yield criterion. (**E**,**F**) Isotropic and anisotropic cortical bone respectively, for maximum compression strain yield criterion. The coefficient of friction is marked as COF.
- Lateral displacement of the implant near the crestal bone for each yield criterion and material model, as shown in Fig. 2.

The results of the simulations were evaluated with respect to the following “failure” criteria:

- Influence of the yield criterion for the cortical bone, namely Tresca, maximum compression stress, and maximum compression strain. A comparison between the isotropic cortical bone model and the anisotropic cortical bone model was performed, for the three yield criteria, as shown in Fig. 1.

**Figure 2:**
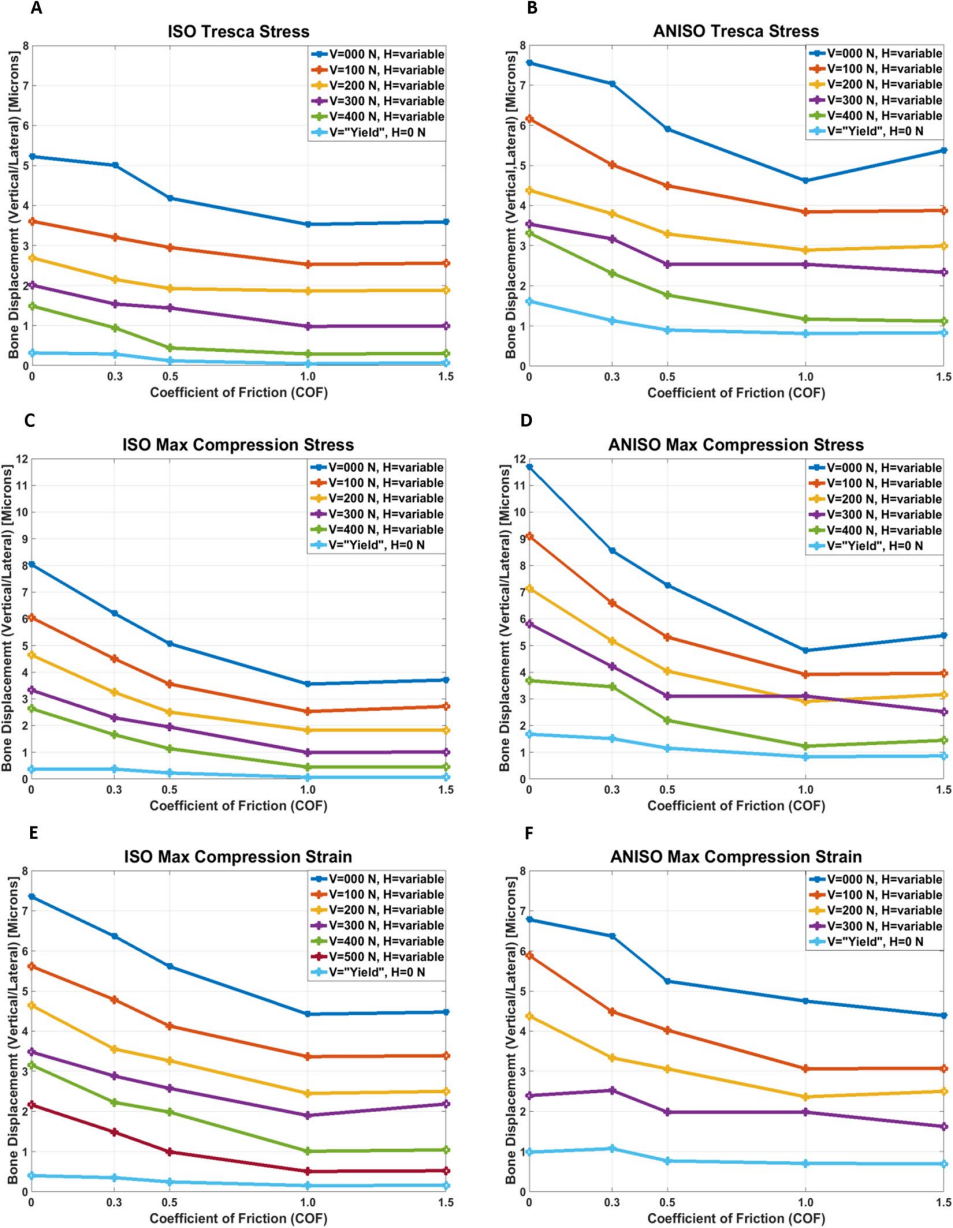
Lateral crestal bone displacement vs. COF for the different loading cases. (**A**,**B**) Isotropic and anisotropic cortical bone respectively, for Tresca stress yield criterion. (**C**,**D**) Isotropic and anisotropic cortical bone respectively, for maximum compression stress yield criterion. (**E**,**F**) Isotropic and anisotropic cortical bone respectively, for maximum compression strain yield criterion.
- Admissible octahedral shear strain levels, according to the mechanostat theory, as shown in Fig. 3.

**Figure 3:**
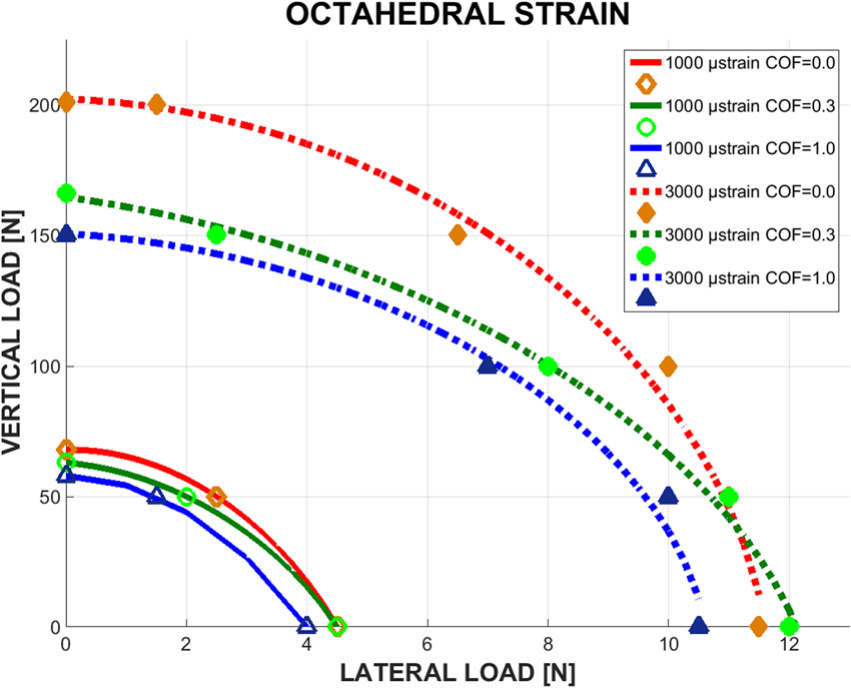
Graphs of lateral load vs. vertical load, causing cortical bone octahedral strain of magnitude 1000 µε (solid curves) and 3000 µε (dashed curves), for 3 levels of COF and isotropic bone model.

Here, it should be emphasized that all these criteria were *first* fulfilled on the bone surface around the implant, denoted in the sequel as the “critical region”, shown in Fig. 8. Therefore, all the results reported in this work represent averaged values over this specific critical region, which spans a roughly 2.2 mm long circular arc of 0.15 mm superficial width.

The results are presented as *failure envelopes* that relate the applied vertical and lateral loads through the selected failure criterion. Those convex envelopes delineate domains of safe, admissible, load combinations.

### Failure envelopes for the cortical bone

From Fig. 1, one can note that the lateral load has a stronger effect on cortical bone yielding than the vertical load, roughly up to 10 times higher. It can also be noted that the failure (yield) locus of the bone is close to an ellipsoid for all the studied cases.

Likewise, when the COF increases, the loads needed to reach yielding decrease, but when the COF is equal or higher than 1, the failure envelopes merge into a single curve. The fully bonded case (µ = ∞) makes an exception to the above, and can be considered as a limit case in which the problem is no longer governed by bone-implant friction, and is therefore of a different mechanical nature.

When comparing the isotropic and anisotropic bone models, the resulting envelopes for the anisotropic case are bigger for the Tresca yield and maximum compression stress criteria, while the opposite is observed for the maximum compression strain yield criterion. In other words, an isotropic bone model is a more conservative kind of model stress-wise.

When comparing the 3 yield criteria chosen in this work, Fig. 1 shows that for the isotropic case, the Tresca yield criterion is the most conservative, followed by maximum compression stress and then strain. For the anisotropic bone model, both Tresca and maximum compressive strain criteria are equivalent, being both more conservative than the maximum compressive stress criterion.

### Crestal bone lateral displacements

The lateral displacement component in the critical region of the crestal bone (see Materials and Methods) was obtained for each studied case, as shown in Fig. 2. In order to ensure primary stability of the implant, this lateral displacement component should be kept as small as possible.

From Fig. 2, a first general observation is that the crestal lateral displacement decreases with increasing coefficient of friction. If the process of osseointegration can be translated into an increasing coefficient of friction, this result just expresses the obvious fact that the better anchored into the bone, the less the implant will move laterally. Next, the general trend of the curves indicates that the higher the vertical load, the smaller the displacement. However, it must be kept in mind that the vertical and the lateral load components are tied together by the yield criterion of the bone (Fig. 1), so that the higher vertical load also corresponds to a lower lateral component and vice-versa. Yet, all the calculated values do not exceed 12 μm, which for the assumed bone properties is a reasonable value^25^. This observation applies to the various yield criteria examined in the simulations, so that they will not be further ranked as to their degree of conservatism.

### Octahedral shear strain

Figure 3 shows the range of admissible loads that cause an octahedral shear strain in the recommended range of the mechanostat theory, namely 1000–3000 με, so that the bone neither experiences “stress shielding” nor excessive deformations.

In Fig. 3, the same trend can be seen regarding the failure envelopes and COF, where an increase in the COF results in smaller “safe zone” of loads, for both the lower (1000 µε) and upper (3000 µε) limits. Here too, it can be observed that lateral loading is up to 20 times more dangerous than vertical loading. But the most striking observation is that the octahedral shear strain failure envelope, according to the “mechanostat” theory, is far more conservative than the other failure criteria investigated earlier in terms of admissible load levels (comparing Figs 1 and 3).

## Discussion

While many previous reports have assessed the interaction between an implant, loaded at an angle and the jawbone, this work is the first of its kind in which the vertical and lateral load components have been systematically separated first, then re-joined through the cortical bone “failure envelope”, thus allowing for the definition of the admissible range of load components.

Our simulations were carried out for a selected set of bone properties so that the specific reported stress or displacement values are not universal, however the trends reported in that work can reasonably be assumed to be valid for other bone properties, and of course implant geometry. A first important outcome of this work is that the lateral load component is far more influential on the cortical bone yielding than the vertical load, so that it should be kept to a minimum to allow for large vertical mastication loads to be exerted safely. A similar claim can be found in the literature^26, 27^, although it was not substantiated by methodical calculations.

Various yield and/or failure criteria were examined for the cortical bone, that was always the first to meet the “failure criterion” as a result of the very high contrast in the elastic properties of the bone components. Those criteria include stress, strain and lateral displacement considerations. As of today, there is no unanimity on a specific yield criterion, so that one can only rank them in terms of conservatism. Considering stress as the relevant mechanical parameter, the combination of isotropic material model and Tresca yield criterion result in the lowest admissible vertical/lateral load components. However, when the octahedral shear strain is considered in the framework of the mechanostat theory, the present results show that it is far more conservative than all the other failure criteria. It can thus be concluded that the most conservative criterion is octahedral shear strain, while the most “liberal” one is the maximum compressive strain. All the other criteria provide failure envelopes that lie between those two bounds, as a benchmark for future numerical simulations of bone-implant interaction.

Of all the failure criteria, the mechanostat is the only criterion that considers bone evolution rather than its flow or fracture. The admissible limits of octahedral shear strain are quite small and well within the elastic range of the bone, which justifies its conservatism. Yet, from Fig. 3, it appears that the lower bound is almost automatically exceeded since the loads are very small. This, in turn, means that the bone will probably not be exposed to a stress shielding condition, at least for the bone-implant system considered here. On the other hand, one can also note that the upper limit is also comprised of relatively small loads, which indicates now that this limit is likely to be routinely exceeded for usual mastication loads. Here, it is important to remind that the (critical) region of interest, where the criteria were always fulfilled first, is located on the bone surface shown (see loading procedures). Incidentally, when bone recess (peri-implantitis) occurs, it does so precisely in this region to start, a fact which might be related to the excessive local strain levels, as per the mechanostat model. From the dental perspective, it can be remarked that today, implant survival is predictable, regardless of the implant geometry, bone quality, surgical and prosthetic treatment protocol. As a result, one of the implant manufacturers’ goal is to achieve the lowest bone loss rate over time, and a stable crestal bone level (bone level around the implant neck). The contribution of stresses and strains to crestal bone level and stability has not been previously considered in the context of both bone quality and bone implant contact stability (COF). The failure envelope concept developed here can be used as a powerful tool to evaluate the effect of the implant geometry on the peri-implant stress and strain fields, in order to add to the crestal bone level stability over time.

Another interesting, albeit counterintuitive, result is that as the coefficient of friction increases, which is deemed here to represent the progressive osseointegration process, the failure envelope shrinks. Stated otherwise, the firmer the bone-implant bonding, the lower the admissible loads. This observation can be rationalized by considering that as long as the implant can slide “independently” of the neighboring bone, the latter will experience lower stress levels. However, as the degree of bonding becomes stronger, the implant is increasingly transferring its load to the neighboring bone, thereby bringing it to yield earlier.

The next important outcome of this work was that of the implant lateral (horizontal) displacements in the crestal bone region. Here, irrespective of the constitutive assumptions and yield criterion, it was found that this component of the displacement is rather small (not exceeding 12 μm), which complies with reported clinical values, of the order of 100 micron^25^. This applies of course up to bone yielding, and the application of higher loads is naturally expected to cause larger displacement values.

Finally, it is believed that the failure envelope approach presented here can be applied to more complex cases of multiple implants or interaction with adjacent natural teeth, that were not considered here.

## Conclusion

The dental-implant bone system has been characterized in terms of a failure envelope that delineates admissible load and strain levels, thereby avoiding crestal bone yielding or under/over straining.

This separate consideration of the vertical and lateral load components demonstrates the potentially more damaging influence of the latter on crestal bone yielding.

The most conservative criterion is octahedral shear strain criterion, while the most “liberal” one is maximum compressive strain criterion, thereby setting bounds on the admissible load levels of dental implants.

The failure envelope shrinks when the bone-implant coefficient of friction increases (e.g. as a result of osseointegration). This indicates a higher efficiency of the load transfer mechanism from the loaded implant to the bone, reducing stress shielding of the bone.

The lateral displacements of the implant have been calculated for all the selected criteria and bone models, and were found to remain well within desired clinical limits up to bone yielding, thereby promoting osseointegration.

According to the mechanostat-recommended strain values, the present results show that the applied strains almost automatically satisfy active bone loading, thereby precluding stress shielding effects. This criterion is also likely to be exceeded, under normal mastication loads, in the critical region of the bone-implant interface, which might account for the initiation of bone resorption.

## Materials and Methods

A mandibular bone section and implant system were modelled using finite element (FE) simulations, to investigate the stress and strain distributions within the bone until yield. Emphasis was put on the separate effect of lateral loading (buccal-lingual, BL direction), versus vertical loading (apical-coronal, AC direction) on the yielding of the bone, within assumptions of linear elasticity. 3D static nonlinear analyses were carried out using the commercial finite element package Abaqus^28^. The selected implant system is closely inspired by a commercial one (MIS Seven implant, http://www.misimplants.com/implants/brands/seven/seven-mf7-13375.html) with a matching abutment and connecting screw from this manufacturer. The bone section geometry was taken from the literature^29^.

### Model Parts

#### The implant system

**Figure 4:**
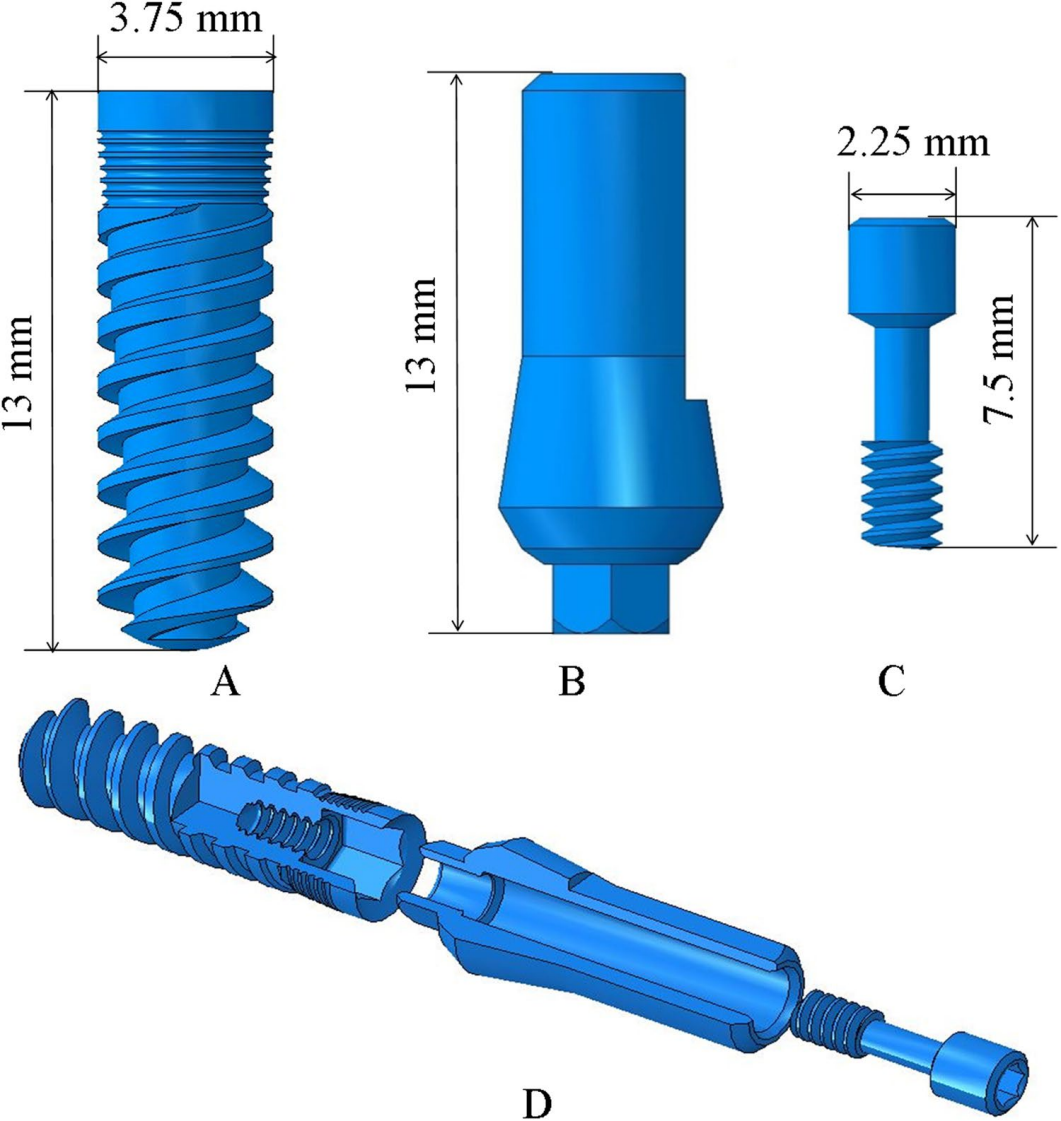
The implant system: (**A**) Implant (**B**) Abutment (**C**) Connecting screw (**D**) Exploded view of the implant system used in the model.

*Implant* (Fig. 4A): The implant has a 3.75 mm diameter and 13 mm length, and it features 5 micro-rings on the neck, and a general conical shape with threads that increase in thickness.

*Abutment* (Fig. 4B): The abutment holds the crown, and it is therefore directly loaded during mastication. The abutment has a 3.75 mm diameter and is 13 mm in length, with a hexagonal connecting section that is inserted to the implant.

*Connecting Screw* (Fig. 4C): The connecting screw holds the abutment and implant together, not allowing any relative movement between them. The screw is 7.5 mm in length.

### The bone system

#### Mandible

**Figure 5:**
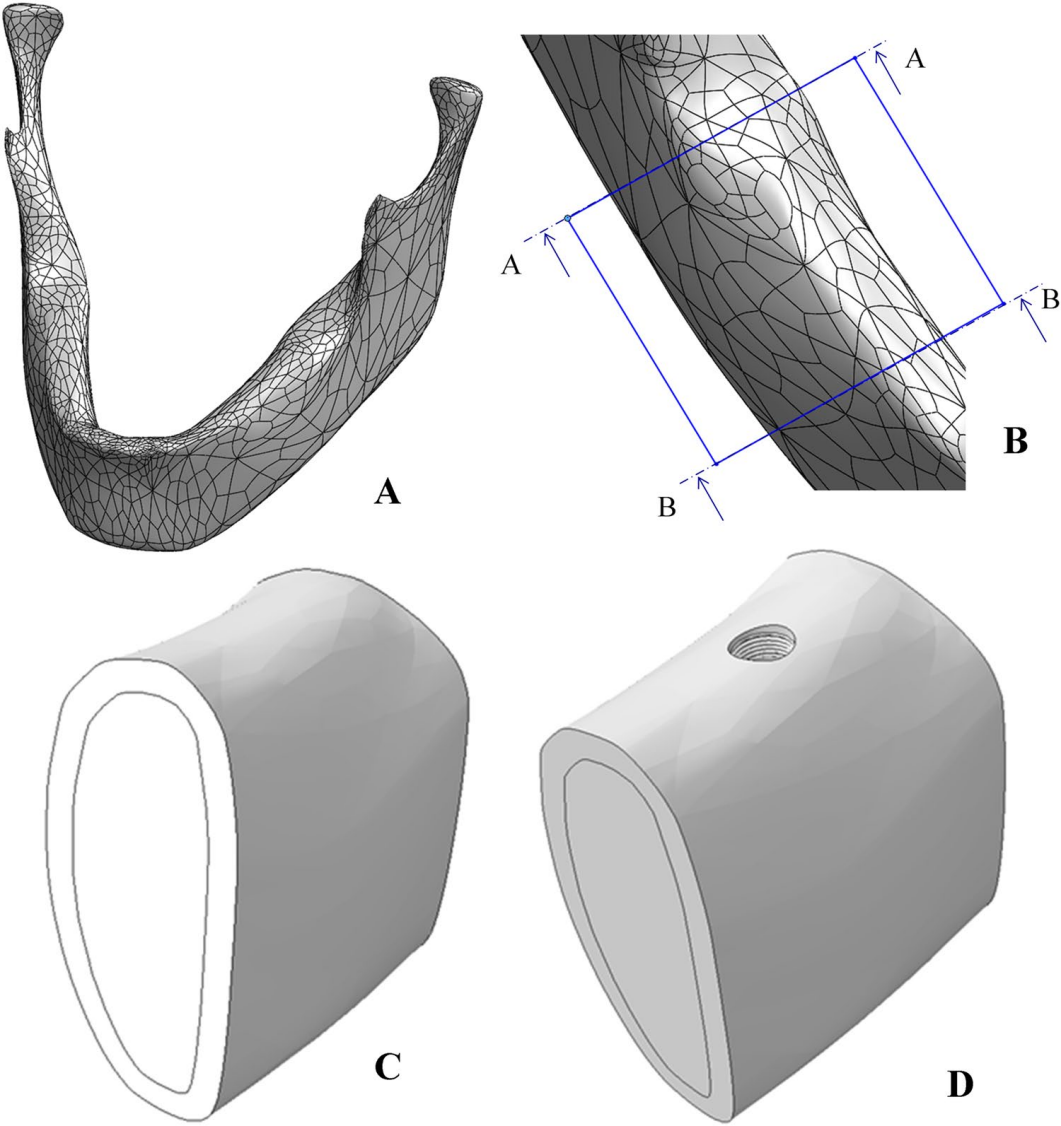
(**A**) Full model of the scanned mandible. (**B**) Cut section of the mandible between the left first and third molars. (**C**) Sectioning the cut bone into cortical and cancellous bone. (**D**) The mandible bone section after implant insertion.

To model the mandible bone section, we started with a fully scanned 3D model of a mandible^29^ as shown in Fig. 5A. Next, a section was cut, between the first and third molars of the left side, as shown in Fig. 5B. Two different bone macro-structures were distinguished: The 2 mm thick *cortical bone shell* ^30^ that surrounds the *internal cancellous bone*, as shown in Fig. 5C.

Next, the implant was inserted into the second molar position in the bone, and a section was extracted, thus providing a perfect geometrical fit between the extracted section and the implant’s geometry, as shown in Fig. 5D.

### Material Properties

A linear elastic, homogenous constitutive model was assumed for both the implant and the bone components.

For the implant system (implant, abutment and connecting screw), isotropic mechanical properties of Ti-6Al-4V ELI^31^ were assigned (Table 1).

**Table 1:** Elastic mechanical properties of materials used in the FE model^17, 19, 31, 32^.

Material	Young’s Modulus E [GPa]	Poisson’s ratio ʋ
Ti-6Al-4V ELI	113.8	0.33
Isotropic Cortical Bone	19.7	0.3
Cancellous bone	0.056	0.34

The *cancellous* bone was assumed to be isotropic, with mechanical properties according to^19, 32^ (Table 1).

**Figure 6:**
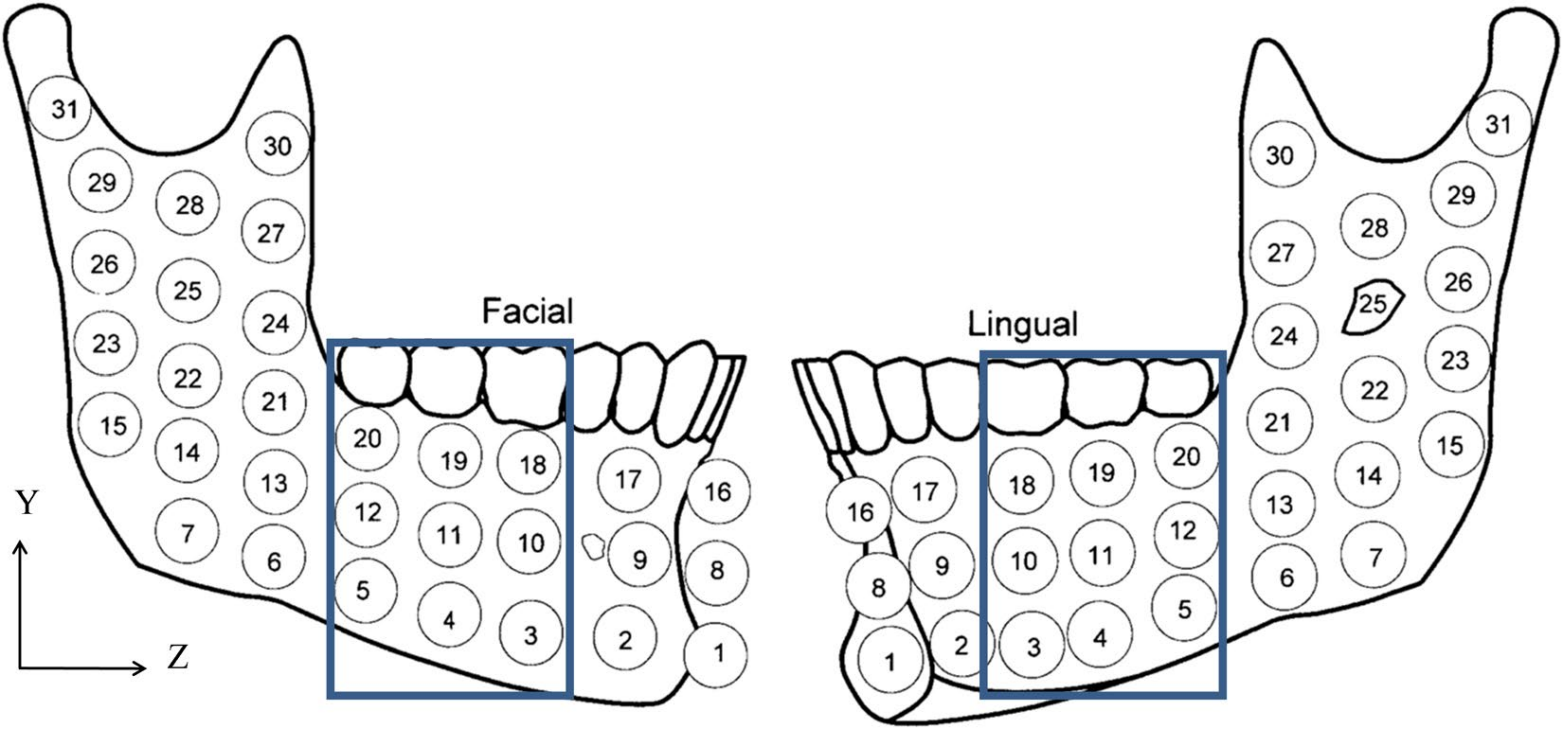
Mandible containing samples (9 × 2) of interest for mechanical properties^17^, the blue rectangle indicates the bone section used in the model. Reprinted with permission from ref. 17.

Two constitutive models were considered for the *cortical* bone: a simplified isotropic model, and an anisotropic model. The mechanical properties of the isotropic cortical model are listed in Table 1. For the anisotropic bone model, the assumption of an orthotropic constitutive model was made according to experimental data reported in ref. 17. In this work, we looked at the section highlighted in Fig. 6, where each of the 9 × 2 different samples has different principal axes and mechanical properties. Since the interest of this work is up to the yield point of the cortical bone, we only considered sample 19 (Fig. 6) from the facial and lingual cortices, located in the immediate vicinity of the implant. An average of the mechanical properties of those 2 samples is needed, and to do so, both of them which are expressed in the local sample coordinate system need to be transformed and expressed in the global coordinate system marked X,Y,Z in Fig. 6. The detailed transformation procedure can be found the Supplementary Information section.

### Mesh

**Figure 7:**
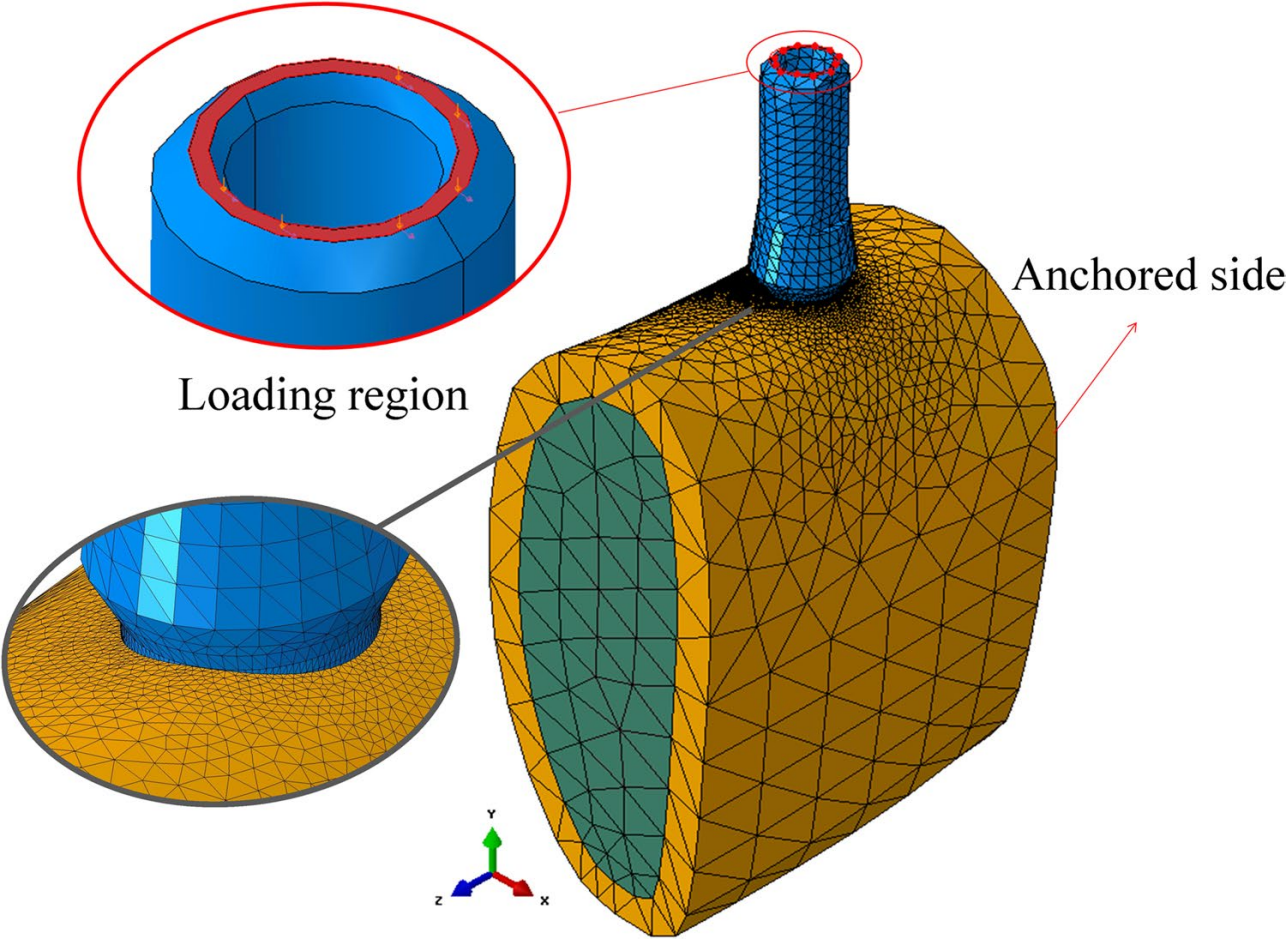
Meshed model of the bone-implant system, showing the refined peri-implant mesh, boundary conditions and loading region in the model.

A mesh of C3D4^28^ tetrahedral elements was created for all the parts of the model. The typical element size for the implant and connecting screw was 0.2 mm, with refinement around the implant neck. Since the abutment was of no particular interest, a coarser mesh of typical element size 0.6 mm was used. For the bone, the mesh used far from the implant insertion hole was of a typical element size of 1.5 mm, while around the implant hole a 0.2 mm typical element size was used. This was to improve the accuracy in the region of interest (crestal bone around the implant). The model comprised of 560386 linear tetrahedral elements in total, the total mesh is presented in Fig. 7.

### Interactions, Boundary conditions, Loads, Failure Criteria

#### Interactions

All parts in the model were assigned frictional contact (Coulomb friction) interaction between them. For the implant system (abutment, implant and connecting screw) a coefficient of friction (COF) for Ti-Ti was assigned (µ = 0.36), according to ASTM-G98^33^.

In order to model the bone-implant interactions, varying from none to fully bonded, the COF was changed systematically, from: µ = 0.0, µ = 0.3, µ = 0.5, µ = 1.0, µ = 1.5 to µ = ∞ (fully bonded).

#### Boundary conditions

The side connected to the Ramus was chosen to be fixed, while the other side was chosen to be left free (Fig. 7). This is justified by the fact that the bone section chosen here is large enough with the implant inserted in its middle, so that the remote boundary conditions do not affect the local results while providing the required constraint for rigid body motion.

#### Loading procedure

The loads applied in the model simulate mastication, therefore they were applied on top of the abutment as shown in Fig. 7. It should be noted here that the reported range and direction of mastication loads exhibits a large variability^26, 34, 35^. In order to investigate the effect of the lateral and vertical loads the following procedure was adopted: step 1: a fixed vertical load was applied on the top of the abutment. Step 2: the vertical load was kept, and a lateral load was gradually applied in the lingual (-x) direction, up to the point of cortical bone yielding. The range of vertical loads was: 0 [N], 100 [N], 200 [N], 300 [N], 400 [N], 500 [N]. One additional case consisted of vertical load only.

All in all, the study comprised 84 simulations $(\mathop{\underbrace{2}}\limits_{Cortical\,{\rm{B}}one}\times \mathop{\underbrace{6}}\limits_{COF}\times \mathop{\underbrace{7}}\limits_{Fixed\,Load}=84)$

#### Failure criteria

**Figure 8:**
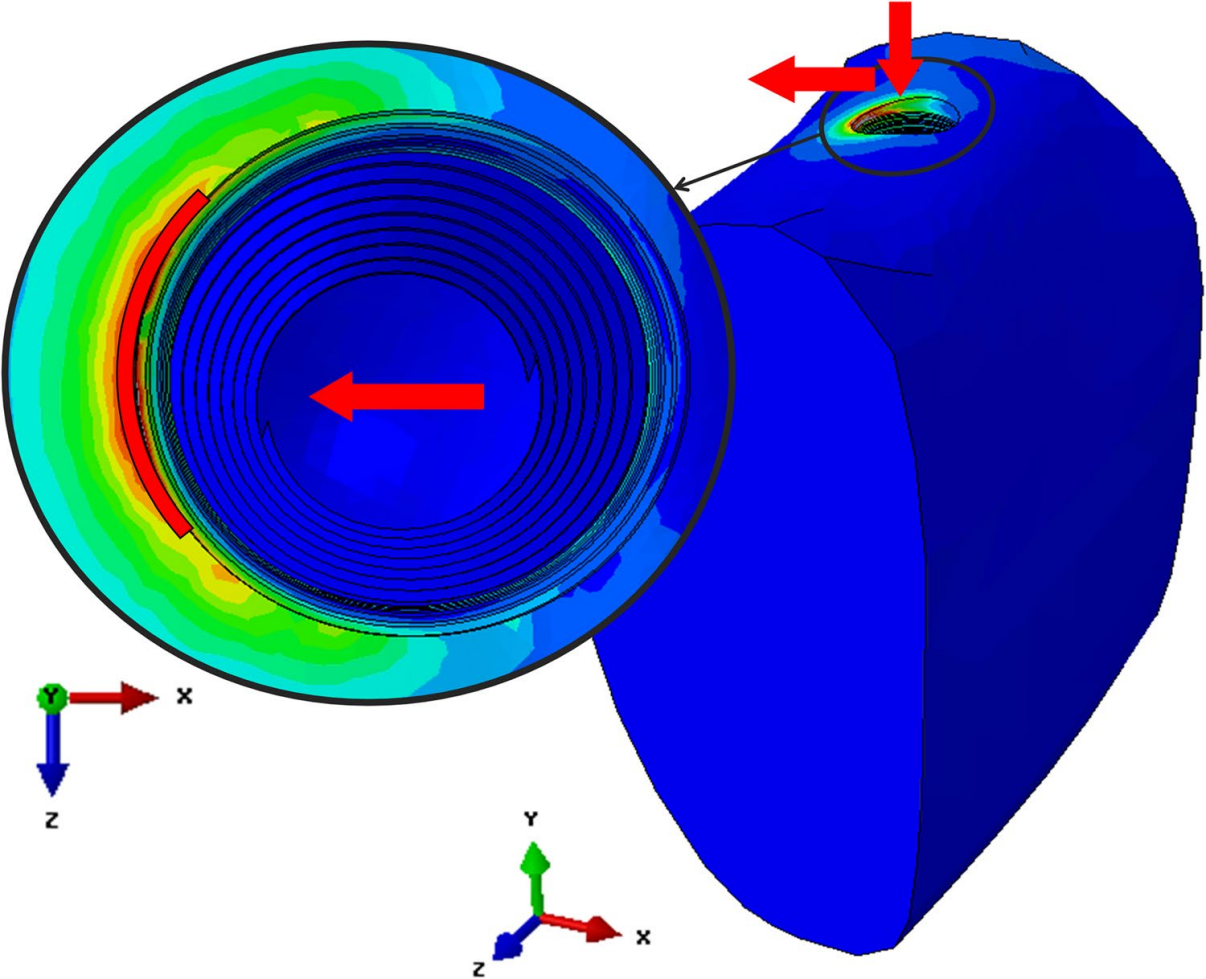
The critical region (marked in red) where the yield criteria were fulfilled. The arrows indicate the loading directions.

To find the yield envelope, 3 *yield* criteria were considered; (1) maximum compression stress with respect to *σ*_*ycompression*_ = 150 *MPa* ^36^, (2) maximum compression strain with respect to ${\varepsilon }_{yCompression}=\frac{{\sigma }_{yCompression}}{E}=0.761 \%$, (3) Tresca stress ${\sigma }_{yTresca}=\frac{{\sigma }_{yCompression}-{\sigma }_{yTension}}{2}=135\,MPa$ ^36^. These criteria were verified through averaging values on a region of interest (critical region) in the immediate vicinity of the implant insertion hole (Fig. 8). This region is a circular arc, approximately 2 mm long, 0.15 mm wide (x), and 0.1 mm deep (y).

Surface averaging was adopted here in order to avoid local peak values of calculated stresses or strains, that may always arise from a single “poorly behaved” element (e.g. excessively distorted), and thus be considered as a numerical artefact.

In addition, the x-direction displacements of the bone in the critical region were measured at the yield point for each model.

In addition, the average octahedral shear strain $({\varepsilon }_{oct}=\frac{2}{3}\sqrt{{({\varepsilon }_{1}-{\varepsilon }_{2})}^{2}+{({\varepsilon }_{2}-{\varepsilon }_{3})}^{2}+{({\varepsilon }_{3}-{\varepsilon }_{1})}^{2}})$ was calculated for the above-mentioned critical region, for the isotropic cortical bone model only. The criterion used here relied on the ranges of strain for which bone homoeostasis is maintained with no bone atrophy, and where bone modelling occurs, i.e. 1000 µɛ–3000 µɛ, thus defining a safe domain of loads.

## Electronic supplementary material

Supplementary Information
