# Supplementary material for: The Failure Envelope Concept Applied To The Bone-Dental Implant System
Source: Sci Rep. 2017 May 17;7:2051. doi: 10.1038/s41598-017-02282-2 (PMC5435678; doi:10.1038/s41598-017-02282-2)
Supplement: Supplementary file 1 — Supplementary Information [file 41598_2017_2282_MOESM1_ESM.pdf]

THE FAILURE ENVELOPE CONCEPT APPLIED TO THE BONE-DENTAL IMPLANT SYSTEM

R. Korabi, K. Shemtov-Yona, A. Dorogoy, and D. Rittel (*)

Faculty of Mechanical Engineering

Technion, 32000 Haifa, Israel

Supplementary Information: Transformation of local stiffness matrix to global coordinates

This section details the transformation, of the bone samples' coordinates from their local coordinate system $\left(\hat{n},\ \hat{t},\ \hat{s}\right)$ to the global model coordinate system $\left(\hat{X},\ \hat{Y},\ \hat{Z}\right)$.

In Dabney et al.[1], cylindrical samples of bone were extracted from the face of the mandible, thus they were normal to the surface. Moreover, each sample had its own principal axis $\left(\hat{n},\ \hat{t},\ \hat{s}\right)$, for which the orthotropic mechanical properties were determined accordingly. In order to average the mechanical properties of the samples of interest, their local coordinate systems must be transformed into the global coordinate system of the whole model.

Given a point $\left(x_0,\ y_0,\ z_0\right)$ in the global coordinate system, on the surface of the bone model (Supplementary Figure 1), where the orthotropic mechanical properties in the local principal axes $\left(\hat{n},\ \hat{t},\ \hat{s}\right)$, are known.

1. The normal to the surface $\hat{n}$ at the point was acquired, and represented in the global coordinate system $\left(\hat{X},\ \hat{Y},\ \hat{Z}\right)$. This normal represents the loading plane, and one of the principal axes [1] (Supplementary Figure 1).

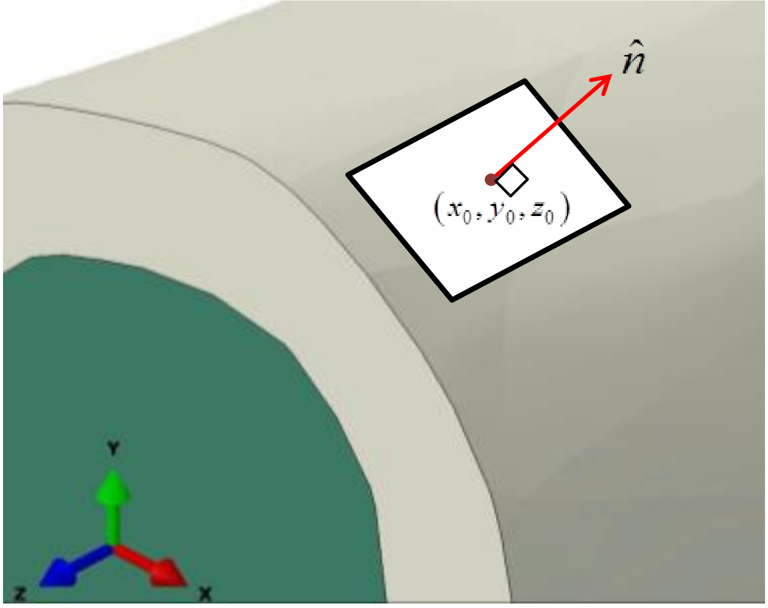

Supplementary Figure 1: Normal to the bone surface for a given point.

2. A projection of the global $\hat{Z}$ - axis to the sample plane was created by a cross product between $\hat{n}$ and the normal to XZ plane $\hat{e}_2 = (0 \quad 1 \quad 0)$, $\hat{n} \times \hat{e}_2 = \hat{L}_z$ (Supplementary Figure 2).
3. The cross product between $\hat{L}_z$ and $\hat{n}$ yields the third orthogonal (perpendicular to $\hat{n}$, $\hat{L}_z$) vector $\hat{L}_y = \hat{L}_z \times \hat{n}$ (Supplementary Figure 2). This results in an orthogonal coordinate system on the sample surface, represented in the global system $(\hat{X}, \ \hat{Y}, \ \hat{Z})$.

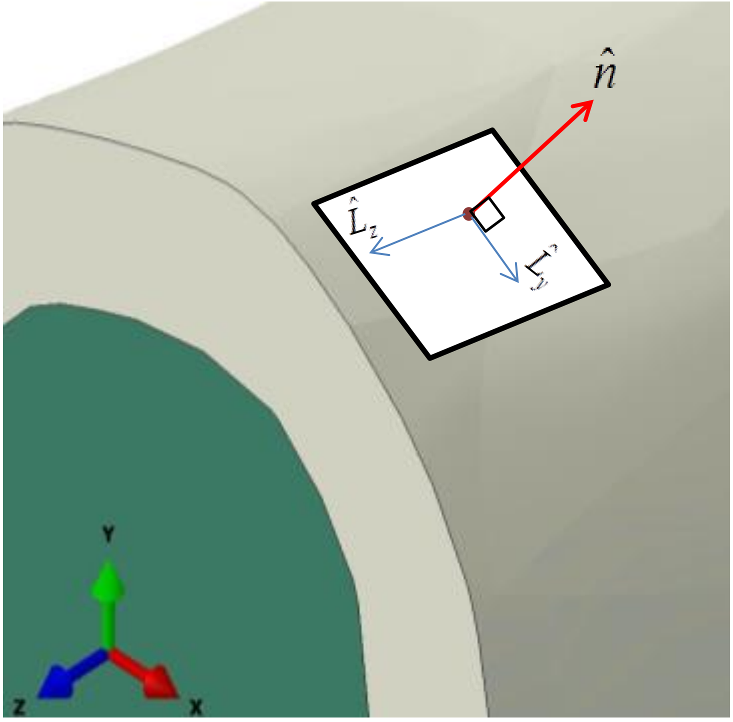

Supplementary Figure 2: Added orthogonal coordinate system on the sample surface

4. According to Dabney et al.[1], the orthotropic mechanical properties of each sample are provided in the maximum stiffness direction, defined here as $\hat{s}$, which was determined using the angle of maximum stiffness $(\hat{\theta})$, also provided in Dabney et al.[1]. $\hat{\theta}$ is the angle between $\hat{s}$ and $\hat{L}_z$ so that its $\hat{L}_y$ and $\hat{L}_z$ components are determined. A cross product between $\hat{n}$ and $\hat{s}$ yields $\hat{t}$. Thus the sample principal axes are now determined in the global coordinate system (Supplementary Figure 3).

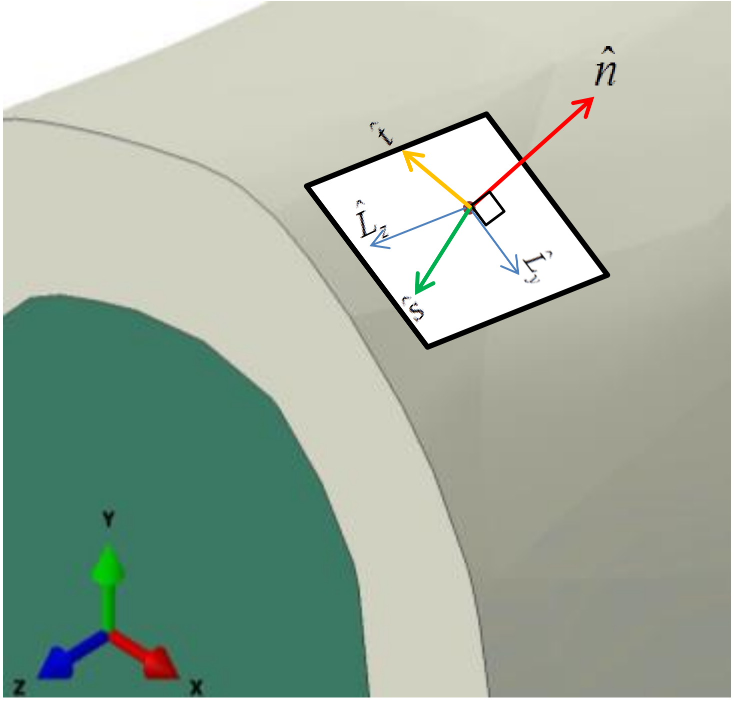

Figure 3: Principal axes obtained in the global coordinate system

5. The vectors $(\hat{n}, \ \hat{t}, \ \hat{s})$ were arranged as the columns of a matrix $A = (\hat{n} \ \ \hat{t} \ \ \hat{s}) = \begin{pmatrix} \hat{n}_x & \hat{t}_x & \hat{s}_x \\ \hat{n}_y & \hat{t}_y & \hat{s}_y \\ \hat{n}_z & \hat{t}_z & \hat{s}_z \end{pmatrix}$. ***A*** is the transformation matrix from the local coordinate system (maximum stiffness) to the global model coordinate system.

6. A was used according to Ting et al.[2], to obtain a transformation matrix K for the stiffness matrix displayed in contracted notation C.

$$K_1 = \begin{bmatrix} A_{11}^2 & A_{12}^2 & A_{13}^2 \\ A_{21}^2 & A_{22}^2 & A_{23}^2 \\ A_{31}^2 & A_{32}^2 & A_{33}^2 \end{bmatrix} \quad , \quad K_2 = \begin{bmatrix} A_{12}A_{13} & A_{13}A_{11} & A_{11}A_{12} \\ A_{22}A_{23} & A_{23}A_{21} & A_{21}A_{22} \\ A_{32}A_{33} & A_{33}A_{31} & A_{31}A_{32} \end{bmatrix}$$

$$K_3 = \begin{bmatrix} A_{21}A_{31} & A_{22}A_{32} & A_{23}A_{33} \\ A_{31}A_{11} & A_{32}A_{12} & A_{33}A_{13} \\ A_{11}A_{21} & A_{12}A_{22} & A_{13}A_{23} \end{bmatrix}$$

$$K_4 = \begin{bmatrix} A_{22}A_{33} + A_{23}A_{32} & A_{23}A_{31} + A_{21}A_{33} & A_{21}A_{32} + A_{22}A_{31} \\ A_{32}A_{13} + A_{33}A_{12} & A_{33}A_{11} + A_{31}A_{13} & A_{31}A_{12} + A_{32}A_{11} \\ A_{12}A_{23} + A_{13}A_{22} & A_{13}A_{21} + A_{11}A_{23} & A_{11}A_{22} + A_{12}A_{21} \end{bmatrix}$$

7. K was used on the the local orthotropic principal stiffness matrix $C_{principal}$ (9 different constants) of each sample given in Dabney et al.[1], to derive the global anisotropic stiffness matrix C_{Global} (21 different constants) $C_{Global} = KC_{principal}K^T$.
8. An average on the desired samples stiffness matrix was calculated, resulting in the sought after mechanical properties of the anisotropic cortical bone.

Bibliography

1. Schwartz-Dabney, C. L. & Dechow, P. C. Variations in cortical material properties throughout the human dentate mandible. *Am. J. Phys. Anthropol.* **120,** 252–277 (2003).
2. Ting, T. C. T. *Anisotropic elasticity: theory and applications*. (Oxford University Press, 1996).
